# Supplementary material for: Protein Maps for Durum Wheat Precision Harvest and Pasta Production
Source: Plants (Basel). 2022 Nov 17;11(22):3149. doi: 10.3390/plants11223149 (PMC9695900; doi:10.3390/plants11223149)
Supplement: Supplementary file 1 [file plants-11-03149-s001.zip › plants-2024766-supplementary.pdf]

Supplementary Figure S1. Images of pasta prepared from semolina with low (<13%; panel A) and high (>13%; panel B) protein content

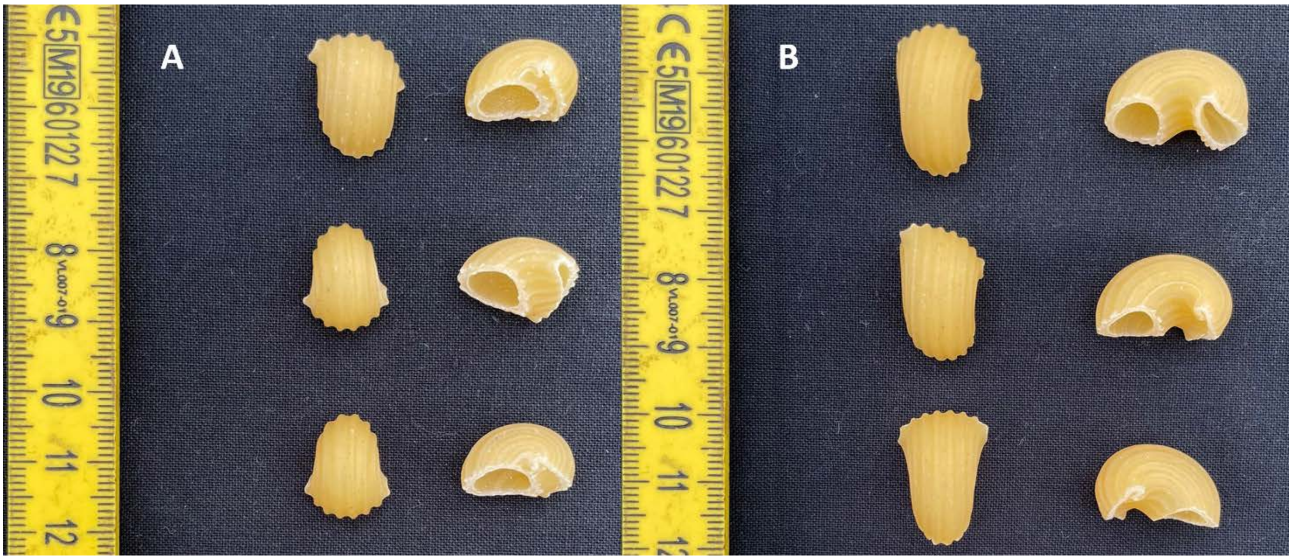

Table S1: Correlation matrices for different protein contents and classes of gluten proteins in different fertility zones**.** Values in red are significant at p<0.05

Total proteins							
	GLI	HMW-GS	LMW-GS	total_GS	GLU/GLI	tot prot_extractable	prot_NIR
GLI	1,000,000	-0.09056	0.065541	0.014448	-0.41994	0.931058	0.773277
HMW-GS	-0.09056	1,000,000	0.134106	0.558534	0.550849	0.119981	0.253582
LMW-GS	0.065541	0.134106	1,000,000	0.896892	0.76234	0.387962	0.395051
total_GS	0.014448	0.558534	0.896892	1,000,000	0.883945	0.378286	0.443844
GLU/GLI	-0.41994	0.550849	0.76234	0.883945	1,000,000	-0.06621	0.060319
tot prot_e	0.931058	0.119981	0.387962	0.378286	-0.06621	1,000,000	0.877851
prot_NIR	0.773277	0.253582	0.395051	0.443844	0.060319	0.877851	1,000,000
Medium Fertility Zones MFZ							
	GLI	HMW-GS	LMW-GS	total_GS	GLU/GLI	tot prot_extractable	prot_NIR
GLI	1,000,000	-0.13317	0.225952	0.104023	-0.40546	0.93963	0.807372
HMW-GS	-0.13317	1,000,000	0.03414	0.60189	0.621245	0.086717	0.251842
LMW-GS	0.225952	0.03414	1,000,000	0.818662	0.621896	0.485891	0.568057
total_GS	0.104023	0.60189	0.818662	1,000,000	0.853897	0.438078	0.598614
GLU/GLI	-0.40546	0.621245	0.621896	0.853897	1,000,000	-0.072684	0.149457
tot prot_e	0.93963	0.086717	0.485891	0.438078	-0.07268	1,000,000	0.935693
prot_NIR	0.807372	0.251842	0.568057	0.598614	0.149457	0.935693	1,000,000
High Fertility Zones HFZ							
	GLI	HMW-GS	LMW-GS	total_GS	GLU/GLI	tot prot_extractable	prot_NIR
GLI	1,000,000	0.219288	0.192207	0.269331	-0.0945	0.951329	0.859132
HMW-GS	0.219288	1,000,000	-0.00964	0.426785	0.368014	0.326288	0.271874
LMW-GS	0.192207	-0.00964	1,000,000	0.900199	0.851642	0.454353	0.383141
total_GS	0.269331	0.426785	0.900199	1,000,000	0.930491	0.553013	0.464912
GLU/GLI	-0.0945	0.368014	0.851642	0.930491	1,000,000	0.216003	0.168726
tot prot_e	0.951329	0.326288	0.454353	0.553013	0.216003	1,000,000	0.892044
prot_NIR	0.859132	0.271874	0.383141	0.464912	0.168726	0.892044	1,000,000
Low Fertility Zones LFZ							
	GLI	HMW-GS	LMW-GS	total_GS	GLU/GLI	tot prot_extractable	prot_NIR
GLI	1,000,000	-0.15671	-0.08483	-0.12526	-0.53369	0.932676	0.776287
HMW-GS	-0.15671	1,000,000	0.236616	0.53422	0.533758	0.040939	0.240296
LMW-GS	-0.08483	0.236616	1,000,000	0.947745	0.816476	0.261595	0.242127
total_GS	-0.12526	0.53422	0.947745	1,000,000	0.885638	0.241044	0.289565
GLU/GLI	-0.53369	0.533758	0.816476	0.885638	1,000,000	-0.200068	-0.102
tot prot_e	0.932676	0.040939	0.261595	0.241044	-0.20007	1,000,000	0.864658
prot_NIR	0.776287	0.240296	0.242127	0.289565	-0.102	0.864658	1,000,000
